# Supplementary material for: Dialysis capacity and nutrition care across Bangladesh: A situational assessment
Source: PLoS One. 2023 Sep 21;18(9):e0291830. doi: 10.1371/journal.pone.0291830 (PMC10513204; doi:10.1371/journal.pone.0291830)
Supplement: S1 Table — This is the S1 Table legend: aBangladesh is divided into 8 administrative divisions which are further divided into 64 districts. Data for the individual districts can be found in S2 Table. bTotal number of centers. cNumber of centers that are Private, NGO or Government operated. An additional 21 centers were not active during the data collection period (14 in Dhaka, 4 in Chittagong, 2 in Rajashahi and 1 in Mymensingh). dThe sum of the population of each division as reported in the 2011 decennial national census. The 2021 national census has been delayed due to Covid-19. Current total population of Bangladesh as of Feb 2022 is ~ 167 million. (DOCX) [file pone.0291830.s002.docx]

**Supporting information**

**S1 Table. Distribution of DFs across Bangladesh based on Geographic Divisions**

| Division^a^ | Total^b^ (P/NGO/G)^c^ | Area (km^2^) (% of total) | Population  (% of total) | Districts with DF | % of division population residing in districts with no DF |
| --- | --- | --- | --- | --- | --- |
| Barisal | 06 (3/2/1) | 13,297 (9.0%) | 8,147,000 (5.7%) | 2 of 6 | 53.3% |
| Chittagong | 38 (32/2/4) | 33,771 (22.9%) | 28,079,000 (19.7%) | 7 of 11 | 13.8% |
| Dhaka | 75 (54/13/8) | 20,551 (13.9%) | 35,881,000 (25.2%) | 9 of 13 | 13.9% |
| Khulna | 14 (9/3/2) | 22,272 (15.1%) | 15,562,000 (10.9%) | 4 of 10 | 42.5% |
| Mymensingh | 05 (3/1/1) | 10,569 (7.2%) | 10,848,000 (7.6%) | 2 of 4 | 32.6% |
| Rajshahi | 10 (4/4/2) | 18,197 (12.3%) | 18,329,000 (12.9%) | 4 of 8 | 36.8% |
| Rangpur | 06 (2/2/2) | 16,317 (11.1%) | 15,665,000 (11.0%) | 2 of 8 | 62.7% |
| Sylhet | 12 (8/2/2) | 12,596 (8.5%) | 9,808,000 (6.9%) | 2 of 4 | 45.9% |
| *Total* | *166 (115/27/24)* | *147,570* | *142,319,000^d^* | *32 of 64* |  |

^a^Bangladesh is divided into 8 administrative divisions which are further divided into 64 districts. Data for the individual districts can be found in S2 Table.

^b^Total number of centers.

^c^Number of centers that are Private, NGO or Government operated. An additional 21 centers were not active during the data collection period (14 in Dhaka, 4 in Chittagong, 2 in Rajashahi and 1 in Mymensingh).

^d^The sum of the population of each division as reported in the 2011 decennial national census. The 2021 national census has been delayed due to Covid-19. Current total population of Bangladesh as of Feb 2022 is ~ 167 million.
