# Supplementary material for: Dialysis capacity and nutrition care across Bangladesh: A situational assessment
Source: PLoS One. 2023 Sep 21;18(9):e0291830. doi: 10.1371/journal.pone.0291830 (PMC10513204; doi:10.1371/journal.pone.0291830)
Supplement: S2 Table — This is the S2 Table legend: 1Data were collected from Population & Housing Census (2011) by Bangladesh Bureau of Statistics (BBS). (DOCX) [file pone.0291830.s003.docx]

**Supporting information**

**S2 Table. Comparison of active number of DFs with population in each district of 8 divisions in Bangladesh.**

| **Serial No** | **District** | **Area**  **(Sq km)^1^** | **Population^1^** | **DFs** | **Population/DF** | **Sq Km/**  **DF** |
| --- | --- | --- | --- | --- | --- | --- |
| **A** | **Barisal** | **13,297** | **8,147,000** | **6** | **1,357,833** | **2216** |
| 1 | Barguna | 1,831 | 882,000 | 0 | N/A | N/A |
| 2 | Barisal | 2,785 | 2,291,000 | 5 | 458,200 | 557 |
| 3 | Bhola | 3,403 | 1,758,000 | 0 | N/A | N/A |
| 4 | Jhalokati | 749 | 596,000 | 0 | N/A | N/A |
| 5 | Patuakhali | 3,221 | 1,517,000 | 1 | 1,517,000 | 3221 |
| 6 | Pirojpur | 1,308 | 1,103,000 | 0 | N/A | N/A |
| **B** | **Chittagong** | **33,771** | **28,079,000** | **38** | **738,921** | **889** |
| 7 | Bandarban | 4,479 | 383,000 | 0 | N/A | N/A |
| 8 | Brahmanbaria | 1,927 | 2,808,000 | 2 | 1,404,000 | 964 |
| 9 | Chandpur | 1,704 | 2,393,000 | 2 | 1,196,500 | 852 |
| 10 | Chittagong | 5,283 | 7,509,000 | 14 | 536,357 | 377 |
| 11 | Comilla | 3,085 | 5,304,000 | 9 | 589,333 | 343 |
| 12 | Cox's Bazar | 2,492 | 2,275,000 | 0 | N/A | N/A |
| 13 | Feni | 928 | 1,420,000 | 5 | 284,000 | 186 |
| 14 | Khagrachhari | 2,700 | 608,000 | 0 | N/A | N/A |
| 15 | Lakshmipur | 1,456 | 1,711,000 | 2 | 855,500 | 728 |
| 16 | Noakhali | 3,601 | 3,072,000 | 4 | 768,000 | 900 |
| 17 | Rangamati | 6,116 | 596,000 | 0 | N/A | N/A |
| **C** | **Dhaka** | **20,551** | **35,881,000** | **75** | **478,413** | **274** |
| 18 | Dhaka | 1,464 | 11,875,000 | 50 | 237,500 | 29 |
| 19 | Faridpur | 2,073 | 1,867,000 | 2 | 933,500 | 1037 |
| 20 | Gazipur | 1,800 | 3,333,000 | 9 | 370,333 | 200 |
| 21 | Gopalganj | 1,490 | 1,149,000 | 1 | 1,149,000 | 1490 |
| 22 | Kishoreganj | 2,689 | 2,853,000 | 1 | 2,853,000 | 2689 |
| 23 | Madaripur | 1,145 | 1,149,000 | 1 | 1,149,000 | 1,145 |
| 24 | Manikganj | 1,379 | 1,379,000 | 0 | N/A | N/A |
| 25 | Munshiganj | 955 | 1,420,000 | 0 | N/A | N/A |
| 26 | Narayanganj | 700 | 2,897,000 | 5 | 579,400 | 140 |
| 27 | Narsingdi | 1,141 | 2,202,000 | 1 | 2,202,000 | 1141 |
| 28 | Rajbari | 1,119 | 1,040,000 | 0 | N/A | N/A |
| 29 | Shariatpur | 1,182 | 1,146,000 | 0 | N/A | N/A |
| 30 | Tangail | 3,414 | 3,571,000 | 5 | 714,200 | 683 |
| **D** | **Khulna** | **22,272** | **15,562,000** | **14** | **1,111,571** | **1591** |
| 31 | Bagerhat | 3,959 | 1,461,000 | 0 | N/A | N/A |
| 32 | Chuadanga | 1,177 | 1,123,000 | 0 | N/A | N/A |
| 33 | Jessore | 2,567 | 2,742,000 | 4 | 685,500 | 642 |
| 34 | Jhenaidah | 1,961 | 1,756,000 | 0 | N/A | N/A |
| 35 | Khulna | 4,394 | 2,294,000 | 5 | 458,800 | 879 |
| 36 | Kushtia | 1,601 | 1,933,000 | 2 | 966,500 | 801 |
| 37 | Magura | 1,049 | 913,000 | 0 | N/A | N/A |
| 38 | Meherpur | 716 | 652,000 | 0 | N/A | N/A |
| 39 | Narail | 990 | 715,000 | 0 | N/A | N/A |
| 40 | Satkhira | 3,858 | 1,973,000 | 3 | 657,667 | 1286 |
| **E** | **Mymensingh** | **10,569** | **10,848,000** | **5** | **2,169,600** | **2114** |
| 41 | Jamalpur | 2,032 | 2,265,000 | 1 | 2,265,000 | 2032 |
| 42 | Mymensingh | 4,363 | 5,042,000 | 4 | 1,260,500 | 1091 |
| 43 | Netrokona | 2,810 | 2,207,000 | 0 | N/A | N/A |
| 44 | Sherpur | 1,364 | 1,334,000 | 0 | N/A | N/A |
| **F** | **Rajshahi** | **18,197** | **18,329,000** | **10** | **1,832,900** | **1820** |
| 45 | Bogra | 2,920 | 3,371,000 | 3 | 1,123,667 | 973 |
| 46 | Joypurhat | 965 | 909,000 | 0 | N/A | N/A |
| 47 | Naogaon | 3,436 | 2,576,000 | 1 | 2,576,000 | 3436 |
| 48 | Natore | 1,896 | 1,696,000 | 0 | N/A | N/A |
| 49 | Chapainawabganj | 1,703 | 1,635,000 | 0 | N/A | N/A |
| 50 | Pabna | 2,372 | 2,497,000 | 0 | N/A | N/A |
| 51 | Rajshahi | 2,407 | 2,573,000 | 4 | 643,250 | 602 |
| 52 | Sirajganj | 2,498 | 3,072,000 | 2 | 1,536,000 | 1249 |
| **G** | **Rangpur** | **16,317** | **15,665,000** | **6** | **2,610,833** | **2720** |
| 53 | Dinajpur | 3,438 | 2,970,000 | 2 | 1,485,000 | 1719 |
| 54 | Gaibandha | 2,179 | 2,349,000 | 0 | N/A | N/A |
| 55 | Kurigram | 2,296 | 2,050,000 | 0 | N/A | N/A |
| 56 | Lalmonirhat | 1,241 | 1,249,000 | 0 | N/A | N/A |
| 57 | Nilphamari | 1,580 | 1,820,000 | 0 | N/A | N/A |
| 58 | Panchagarh | 1,405 | 981,000 | 0 | N/A | N/A |
| 59 | Rangpur | 2,368 | 2,866,000 | 4 | 716,500 | 592 |
| 60 | Thakurgaon | 1,810 | 1,380,000 | 0 | N/A | N/A |
| **H** | **Sylhet** | **12,596** | **9,808,000** | **12** | **817,333** | **1050** |
| 61 | Habiganj | 2,637 | 2,059,000 | 0 | N/A | N/A |
| 62 | Moulvibazar | 2,799 | 1,902,000 | 2 | 951,000 | 1400 |
| 63 | Sunamganj | 3,670 | 2,443,000 | 0 | N/A | N/A |
| 64 | Sylhet | 3,490 | 3,404,000 | 10 | 340,400 | 349 |
| **Total** | **Bangladesh** | **147,570** | **142,319,000** | **166** | **857,343** | **889** |

^1^Data were collected from Population & Housing Census (2011) by Bangladesh Bureau of Statistics (BBS).
