# Supplementary material for: Dialysis capacity and nutrition care across Bangladesh: A situational assessment
Source: PLoS One. 2023 Sep 21;18(9):e0291830. doi: 10.1371/journal.pone.0291830 (PMC10513204; doi:10.1371/journal.pone.0291830)
Supplement: S1 File — (DOCX) [file pone.0291830.s004.docx]

**Supporting information**

**S1 File. The modified 31-Item Questionnaire.**

| This modified 31-Item Questionnaire was used to observe the current Scenario of Bangladeshi Dialysis Facilities which was prepared based on a Malaysian 17-item questionnaire [[1](#_ENREF_1)] and the additional14 questions (shown in blue) were added based on Doctor's and Nutritionist's suggestions.  **Dialysis Capacity and Nutrition Care across Bangladesh**  **Hospital Name:**  No:  Division: District:  **A Survey on Nutrition Practices in Bangladeshi Hemodialysis Facilities** | |
| --- | --- |
| **Section A: Your Center Characteristics** | |
|  | Type of DF:  ☐Government ☐Private ☐Non-Government Organization (NGO) |
|  | Dialysis charge with new dialyzer? |
|  | Dialysis charge with reusing dialyzer? |
|  | Maximum number of times of using a dialyzer? |
|  | Number of shifts in your center?   1. One shift 2. Two shifts 3. Three shifts 4. Four shifts |
|  | Total number of dialysis machine in your center? |
|  | Who are the operators of dialysis machine?(*more than one answer may be chosen*)  ☐ Nurses  ☐ Medical Technicians  ☐ Medical Assistants |
|  | Total number of HD patients at your DF: ____________________ |
|  | Does the DF have access to a nutritionist?  ☐Yes  ☐No |
| **Section B: Nutrition Screening & Education** | |
|  | What is/are the nutrition parameter/s monitored routinely for patients at your center? *(more than one answer may be chosen)*  ☐Body Mass Index  ☐Serum albumin  ☐normalized protein catabolic rate (nPCR)  ☐Dietary intake  ☐Subjective Global Assessment  ☐Malnutrition inflammation score  ☐Others (please state: __________________________________________)  ☐Do not monitor any nutritional parameter. |
|  | At your center, do you provide ongoing nutrition education to patients?  ☐Yes ☐No (*go to question 17 if the answer is no*) |
|  | Who provide/s the nutrition education to your patients? *(more than one answer may be chosen)*  ☐ Doctors  ☐Dietitians  ☐Nurses  ☐Medical assistants  ☐Pharmacists  ☐Others (please state: _____________________________________________) |
|  | Does your centers use any education materials?  ☐ Yes ☐ No, only oral advice |
|  | What form of education material/s is/are used for the nutrition education? *(more than one answer may be chosen)*  ☐Posters  ☐Flipchart  ☐Pamphlet  ☐Booklet  ☐Multimedia (e.g. video, software or apps)  ☐Others (please state: __________________________________________) |
|  | How frequently is nutrition education provided to your patients?  ☐ Regular basic (please state frequency: _____times in a year)  ☐ As per required/referral by doctors  ☐ Others (please state: __________________________________________) |
|  | How is the nutrition counseling delivered to your patients?  ☐ Individual counseling  ☐Group counseling  ☐Both |
| **Section C: Renal Specific Oral Nutrition Supplement (ONS)** | |
|  | Are your patients recommended any renal specific oral nutrition supplement (ONS) on a regular basis?  ☐Yes ☐No (go to question 21 if the answer is no) |
|  | What is/are the indications for recommending renal specific ONS to your patients? __________________ |
|  | Who recommends the use of ONS to your patients? *(more than one answer may be chosen)*  ☐ Doctors  ☐Dietitians  ☐Nurses  ☐Medical assistants  ☐Pharmacists  ☐Patients themselves/their family members  ☐Others (please state: _____________________________________________) |
|  | Does your center provide ONS to the patients?  ☐Yes, free of charge.  ☐Yes, with patients buying.  ☐No, patients buy outside. |
|  | Why is renal specific ONS NOT recommended to your patients who need them?______________________ |
| **Section D: In-center Meal Provision** | |
|  | Are your patients allowed to eat during their dialysis session?  ☐Yes ☐No |
|  | Does your center provide meals to the patients?  ☐Yes ☐No (*go to question 26 if the answer is no*) |
|  | What kind of meals are provided to the patients?  ☐Full meal ☐Light meal |
|  | What are the items name which provided to the patients? ____________________________ |
| **Section E: Miscellaneous** | |
|  | If access to a dietitian, what is the degree of the dietitian? ___________________________ |
|  | Who provide the cost for nutrition parameter checking (i.e. albumin test)?  ☐Patient ☐Hospital |
|  | Approximate Cost for health screening? ___________________________________ |
|  | What is the educational qualification of nurse or medical assistant who provide nutrition education in some hospital? _________________________________ |
|  | For a doctor, does he have any relevant professional nutrition training (i.e. completed a dietetic internship)?  ☐Yes ☐No  Please specify ____________________________________ |
|  | For other health care professionals, do they have any professional nutrition training?  ☐Yes ☐No  Please specify ____________________________________ |
| **Open Comments (if any):** | |

1. Khor B-H, Chinna K, Gafor AHA, Morad Z, Ahmad G, Bavanandam S, et al. The state of nutrition care in outpatient hemodialysis settings in Malaysia: a nationwide survey. 2018;18(1):1-10.
