# Supplementary material for: Dialysis capacity and nutrition care across Bangladesh: A situational assessment
Source: PLoS One. 2023 Sep 21;18(9):e0291830. doi: 10.1371/journal.pone.0291830 (PMC10513204; doi:10.1371/journal.pone.0291830)
Supplement: Attachment — Submitted filename: Responses to Reviewers.docx [file pone.0291830.s005.docx]

**Based on the reviewers’ comments from our previous submission (PONE-D-23-05572)**

*Thank you for your thorough review. I also appreciate the Editor for considering our manuscript as “New Submission” to their prestigious journal. Below is point to point responses to the Reviewers’ comments.*

**Comments to the Author**

**1. Is the manuscript technically sound, and do the data support the conclusion?**

The manuscript must describe a technically sound piece of scientific research with data that supports the conclusions. Experiments must have been conducted rigorously, with appropriate controls, replication, and sample sizes. The conclusions must be drawn appropriately based on the data presented.

Reviewer #1: Yes

Reviewer #2: Yes

**2. Has the statistical analysis been performed appropriately and rigorously?**

Reviewer #1: Yes

Reviewer #2: Yes

**3. Have the authors made all data underlying the findings in their manuscript fully available?**

The PLOS Data policy requires authors to make all data underlying the findings described in their manuscript fully available without restriction, with rare exception (please refer to the Data Availability Statement in the manuscript PDF file). The data should be provided as part of the manuscript or its supporting information, or deposited to a public repository. For example, in addition to summary statistics, the data points behind means, medians and variance measures should be available. If there are restrictions on publicly sharing data—e.g. participant privacy or use of data from a third party—those must be specified.

Reviewer #1: Yes

Reviewer #2: Yes

**4. Is the manuscript presented in an intelligible fashion and written in Standard English?**

PLOS ONE does not copyedit accepted manuscripts, so the language in submitted articles must be clear, correct, and unambiguous. Any typographical or grammatical errors should be corrected at revision, so please note any specific errors here.

Reviewer #1: Yes

Reviewer #2: No

**5. Review Comments to the Author**

Please use the space provided to explain your answers to the questions above. You may also include additional comments for the author, including concerns about dual publication, research ethics, or publication ethics. (Please upload your review as an attachment if it exceeds 20,000 characters)

**Ans:** Thank you for your comment. We have tried our best to minimize the write up from 36 pages to 27 pages now without avoiding any significant data or information.

**Reviewer #1:**

The study entitled "Dialysis Capacity and Nutrition Care across Bangladesh: A Situational Assessment" was well-written and these are my comments:

1. **Please mention the difference between private and non-governmental organizations and why you didn’t consider them as one group?**

**Ans:** It was observed that there are significant differences in the management of dialysis services across different dialysis facilities in terms of cost, service, nutrition care, and provision of food. To account for these differences, the identified facilities were divided into three sectors; government, private, and non-governmental organizations (NGOs). **Page 5, Lines (99-103)**

2. **What kind of Specific oral nutrition supplement (ONS) did you ask about? Give examples.**

**Ans:** we inquired about the availability of complete nutrition supplements specifically designed for people with kidney disease, which are low in potassium, phosphorus, and sodium, and contain a blend of high proteins, carbohydrates, and fats, such as Nepro, Renalcal, Prorenal, Novasource Renal, and Suplena. **Page: 318, lines (318-321)**

3. **The title of Table 5. "Recommendations to improve future dialysis services" should be revised; it doesn’t provide recommendations but rather limitations.**

**Ans:** Thank you for your comment. It is now modified based on your suggestions.

4. **In the results section; the same data in each table and figure was written again in the results paragraphs, so, avoid repetition by deleting the repeated information.**

**Ans:** Thank you for your comment. However, we did not make any repetition of data in table and figure. We will really appreciate if you could specify this statement.

5. **Try to summarize the conclusion focusing on the key messages from your study**.

**Ans.** There is still no published data regarding dialysis facilities and nutrition care services in Bangladesh. However, in developed nations, adequate nutrition care was proved to be a cost-effective tool in treating dialysis patients. The objective of the study was to get information on the existing services available in the country so that necessary strategies could be taken in the long run.

**Reviewer #2:**

The authors studies dialysis service capacity and nutrition services across Bangladesh. This highlights common problems of the dialysis services in low and low middle income countries.

Below are my comments:

Generally, the study is limited by the unnecessarily detailed discussion of logic facts, and this made the language appear redundant. The overall language of the manuscript needs to be sharp and to the point. This is more pronounced in the discussion section. Examples are:

1- **Page 5: no need to mention the names of 63 districts in Bangladesh in the figure legend.**

**Ans:** Removed; and mention it in the supporting file **table S2**

2. **Table 5 needs to be more concise and targeted.**

**Ans:** Modified accordingly.

3. **The discussion section needs re-writing with omission of the parts not discussing the results e.g. line 408 page 23 discussing Access use in HD initiation.**

**Ans:**

4. The study language needs thorough revision for grammatical errors and language clarity. e.g. the repeated use of the word "correlated" in place of difference between different groups in 211 and 216 page 10 and 239 page 11.

**Ans.** Modified accordingly.

5. Dialyzer is merely the filter and not synonymous for artificial kidney (line 218; page 10).

**Ans.** Modified accordingly.
